# The nomogram predicting the early failure rate of the Pavlik harness for developmental dysplasia of the hip in infants under 6 months of age

**DOI:** 10.3389/fped.2022.1018641

**Published:** 2022-10-03

**Authors:** Pan Gou, Kai Gao, Xiaoting Wang, Xing Liu

**Affiliations:** Orthopedic Center of Children, Chongqing Medical University, Chongqing, China

**Keywords:** 0–6 months, infants, nomogram, developmental dysplasia of the hip, harness

## Abstract

**Background:**

The aim of our study was to develop a nomogram predicting the early failure rate of Pavlik harness in infants under 6 months of age with developmental dysplasia of the hip (DDH).

**Methods:**

We retrospectively analyzed the clinical data of 227 patients (372 hips) with DDH who were treated with Pavlik harness at our institution from August 2019 to January 2022. Fifty-eight patients (102 hips) failed the Pavlik harness treatment, and 169 patients (270 hips) were successfully treated. Then, the independent risk factors for treatment failure were determined *via* univariate and multivariate logistic regression and used to generate the nomogram predicting the failure rate of the Pavlik harness.

**Results:**

It was found that age at initial treatment (OR 1.031, 95% CI 1.022–1.040, *P* < 0.001), angle α (OR 0.723, 95% CI 0.671–0.779, *P* < 0.001), and concomitant deformity (OR 0.129, 95% CI 0.036–0.459, *p* = 0.002) were independent risk factors for treatment failure. The nomogram showed good discrimination [the area under the curve (AUC): 0.862], good calibration, and a net benefit in the range of probabilities between 5 and 90% according to the decision curve analysis.

**Conclusion:**

This study successfully established the nomogram prediction model based on three independent risk factors. Due to the high level of predicting accuracy, this nomogram could be a useful resource for pediatric orthopedic surgeons to identify patients at major risk of Pavlik harness failure who might need more reliable treatments.

## Introduction

Developmental dysplasia of the hip (DDH) is the most common pediatric hip disorder. Pavlik harness has been considered as the first choice for infants aged 0 to 6 months with DDH (1, 2), that is successful in approximately 70–95% of cases (3, 4), but not free of some limitations. First, as a soft harness, it allows the hip to move within a certain range, so parents occasionally complain about the inconvenience of dressing and caring for during treatment (5, 6). Second, the complications consist of femoral nerve palsy (7), residual acetabular dysplasia (8–10), and avascular necrosis of the femoral head (11, 12). Third, several variables are found to be possible risk factors for treatment failure: older age of initial treatment, Graf III/IV, combined other deformities, bilaterality of pathology, male patients, developing femoral nerve palsy, etc. (6, 13, 14).

Generally, owing to increased success rates and fewer complications, the rigid brace could be used as second-line treatment after failure of Pavlik harness in children under 6 months of age (15, 16), and closed reduction and spica casting was a better method for children older than 6 months who failed early harness or brace (17). In this clinical scenario, it appears therefore of great importance to identify the patients at high risk of Pavlik harness failure and take more reliable treatment timely to improve early treatment effects. However, at present, no reliable prediction models exist for the treatment outcomes of Pavlik harness. With this knowledge in mind, we aimed to develop and validate a nomogram for predicting the early failure probability of Pavlik harness based on fundamental, clinical, and ultrasound variables in infants under 6 months of age with DDH.

## Materials and methods

After the research ethics board of our institution approved this research protocol, the ethics reference number is 2022 Ethics Review No. 245 of the Institutional Review Board. We retrospectively analyzed 227 patients (372 hips) who underwent Pavlik harness for DDH at our hospital from August 2019 to January 2022. The exclusion criteria were as follows: (1) confirmed diagnosis of Graf IIa; (2) being aged older than 6 months at the time of diagnosis; (3) treated by brace or spica casting initially; and (4) data deficiency.

In general, after sonographers performed an ultrasound of the hip, orthopedists recommended treatment options. Generally, we suggested to hold patients with Graf IIa hips in flexion and abduction position, because a high proportion of these are physiologically immature which could normalize with growth and without special treatment. For Graf IIb ∼ IV DDH of 0–6 months infants, we chose Pavlik harness as their initial treatment. During the treatment process, it was required to wear the harness for 24 h as much as possible and follow up once every 2 weeks by ultrasound. For Graf IIb, IIc, and IId, if there was no significant improvement in either angle alpha (angle α) or angle beta (angle β) after 2–3 weeks of re-examination, the Pavlik harness would be regarded as failure and was changed to human position braces or spica casting. For Graf III and IV, persistent dislocation during follow-up suggested failure of the Pavlik harness.

About the data collection, age at initial treatment, gender, side of pathology hip, clinical type, ultrasound type, angle α, angle β, concomitant deformity, length of treatment, and follow-up were recorded from all patients. In particular, because it was difficult to measure the angle α and angle β for Graf III/IV DDH, the default values were 43 and 80°, respectively, in this study.

Data analysis and statistical plotting were performed using SPSS version 25.0 statistical software (SPSS corp., Chicago, IL, USA) and R language software (R 3. 6. 0). The critical *p*-value for significance was set at 0.05. The continuous variables which conformed to normal distribution were expressed using mean ± standard deviation (SD), and other continuous variables showing non-normal distribution were expressed as medians (interquartile ranges). The categorical variables were described by frequencies and percentages. Differences of cohort characteristics were assessed with a paired *t*-test, Mann–Whitney test, Pearson chi-square analysis, and Fisher’s exact test. All variables were future assessed using univariate binary logistic regression and collinearity test. After excluding redundant variables with excessive collinearity (VIF >3.0), the remaining variables with *P* < 0.05 were screened into multivariable logistic regression analysis to identify the independent risk factors for Pavlik harness failure.

The nomogram was developed based on the multivariable model through R 3. 6. 0. Receiver operator characteristic curves (ROC) were produced, and the area under the curve (AUC) with the 95% CI was calculated to evaluate the discrimination. Additionally, the nomogram was internally validated using tenfold cross-validation. Calibration plots were obtained, where the x-axis represents the predicted probability and the *y*-axis represents the actual observed accuracy of the model. The calibration was evaluated by comparing the predicted and actual observed probability curves. Finally, decision curve analysis (DCA) evaluated the net benefit of the model.

## Results

A total of 372 hips were enrolled in this study. The average length of follow-up was 14.8 ± 7.4 months (range, 6 to 36 mo). The failure rate of the Pavlik harness was 27.4% (102/372), and then, the cohort characteristics between the success group and the failure group are presented in Table 1. In the failure group, there were older patients at initial treatment (*P* < 0.001), worse clinical type of the hip (*P* < 0.001), and more concomitant deformities (*P* = 0.002). For ultrasound analysis, angle α (*P* < 0.001) and angle β (*P* = 0.008) were associated with significant differences. As expected, the treatment and follow-up duration of the failure group were prolonged. In Table 1, 19 hips were accompanied with other deformities (7/19 deformities presented clubfoot, 6/19 deformities presented congenital muscular torticollis, 4/19 deformities presented cerebral palsy, and 2/19 deformities presented joint contracture).

**TABLE 1:** Comparisons of clinical and imaging characteristics.

	Success group	Failure group	Statistical values	*P*
No. of hips (cases)	270 (169)	102 (58)		
Age at initial treatment (day)	83.0 ± 40.2	102.3 ± 37.6	–4.20	< 0.001*
Gender (hip)			1.43	0.232
Males	30	16		
Females	240	86		
Side of pathology hip (case)			3.92	0.048*
Unilateral	64	18		
Bilateral	95	50		
Clinical type (hip)			57.3	< 0.001*
Dysplasia (IIa/IIb/IIc)	252	63		
Subluxation (D)	8	14		
Dislocation (III/IV)	10	25		
Ultrasound type (hip)			70.8	< 0.001*
Ib	242	50		
IIc	10	13		
D	8	14		
III	10	12		
IV	0	13		
Angle α (°)	57 (54, 58)	50 (43, 56)	–8.52	< 0.001*
Angle β (°)	70 (65, 74)	72 (67, 80)	–2.66	0.008*
Concomitant deformity (hip)			9.34	0.002*
Yes	8	11		
Not	262	91		
Duration of treatment (month)	2.2 ± 1.5	2.6 ± 1.7	–2.37	0.019*
Duration of follow-up (month)	12.2 ± 7.2	19.5 ± 8.1	–8.44	< 0.001*

Continuous variables are expressed as the mean ± standard deviation or medians (interquartile ranges). *Statistically significant.

All the basic information was included in the univariate logistic analysis and collinearity test (Table 2). The result indicated that age, side of pathology hip, clinical type, ultrasound classification, angle α, angle β, and concomitant deformity were significantly associated with treatment failure of Pavlik harness (*P* < 0.05). In addition, there is excessive collinearity (VIF >3.0) among clinical type, ultrasound classification, and angle α. Given that angle α was the firsthand clinical data and largely determined clinical type and ultrasound classification, angle α and other screening factors with *P* < 0.05 were included in subsequent multiple logistic regression analysis (Table 3). The results showed that age at initial treatment (OR 1.031, 95% CI 1.022–1.040, *P* < 0.001), angle α (OR 0.723, 95% CI 0.671–0.779, *P* < 0.001), and concomitant deformity (OR 0.129, 95% CI 0.036–0.459, *P* = 0.002) were independent risk factors for Pavlik harness failure (Table 3).

**TABLE 2:** Univariate logistic regression analysis.

Parameter	Coefficient	*P*	OR	95% CI for OR	VIF	Lower upper
Age at initial treatment	0.012	< 0.001*	1.013	1.006	1.019	1.438
Gender	0.398	0.234	0.672	0.349	1.293	1.035
Side of pathology hip	0.627	0.049*	1.871	1.002	3.496	1.049
Clinical typ	2.159	< 0.001*	8.667	4.648	16.159	3.069
Ultrasound classification	0.428	< 0.001*	1.534	1.285	1.831	5.646
Angle α (°)	–0.206	< 0.001*	0.813	0.775	0.854	3.713
Angle β (°)	0.044	0.012*	1.045	1.010	1.081	1.314
Concomitant deformity	–1.376	0.004*	0.253	0.099	0.648	1.017

**TABLE 3:** Multivariate logistic regression analysis.

Parameter	Coefficient	*P*	OR	95% CI for OR	Lower upper
Age at initial treatment	0.030	< 0.001*	1.031	1.022	1.040
Side of pathology hip	0.638	0.118	0.529	0.237	1.177
Angle α (°)	–0.325	< 0.001*	0.723	0.671	0.779
Angle β (°)	–0.031	0.184	0.969	0.925	1.015
Concomitant deformity	–2.045	0.002*	0.129	0.036	0.459

**FIGURE 1:**
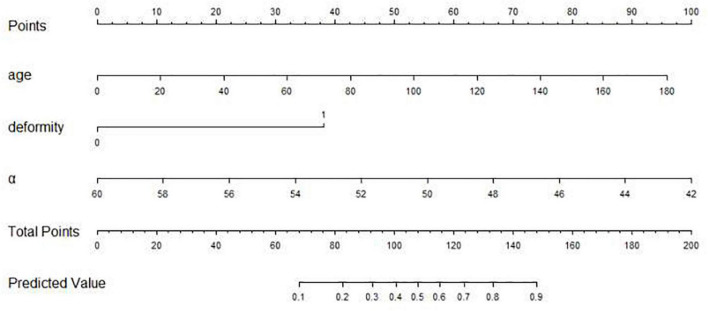
The nomogram for predicting the failure rate of Pavlik harness. Based on age, deformity, and angle α (age: days; deformity: 0: without concomitant deformity, 1: with concomitant deformity; angle α: degrees), we can get a total point, corresponding to the predicted value.

**FIGURE 2:**
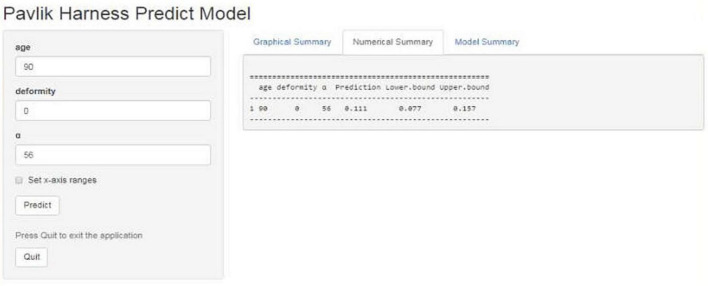
Example of the nomogram. A 90-day patient with developmental dysplasia of the hip, without concomitant deformity, and an angle α of 56° predicted a failure rate of 11.1% (95% CI 0.077–0.157).

As Figure 1 illustrates, the nomogram was made based on the three independent risk factors: age (day), concomitant deformity (without: 0; with: 1), and angle α (deg.). By inputting three variables conveniently, our nomogram prediction model was capable of predicting the failure rate of the Pavlik harness. Figure 2 shows that a 90-day patient with developmental dysplasia of the hip, without concomitant deformity, and an angle α of 56° predicted a failure rate of 11.1% (95% CI 0.077–0.157).

**FIGURE 3:**
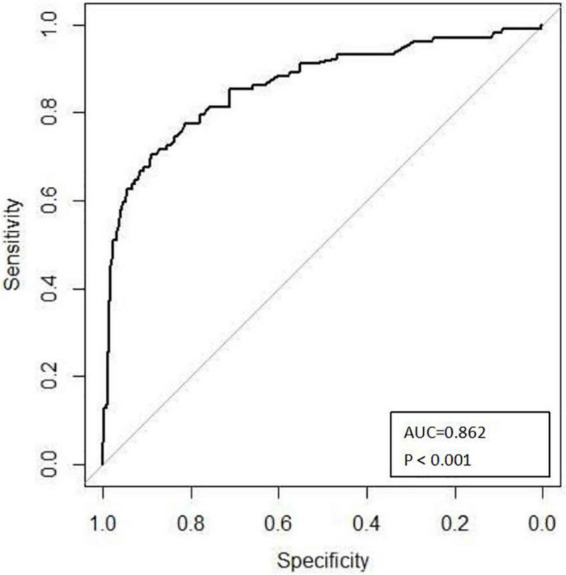
ROC curves to predict the failure rate of the Pavlik harness.

**FIGURE 4:**
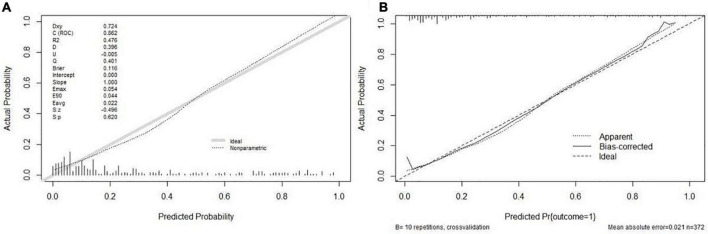
Calibration plots of the nomogram. **(A)** The calibration plot for the modeling cohort; **(B)** the calibration plot for the validation cohort.

**FIGURE 5:**
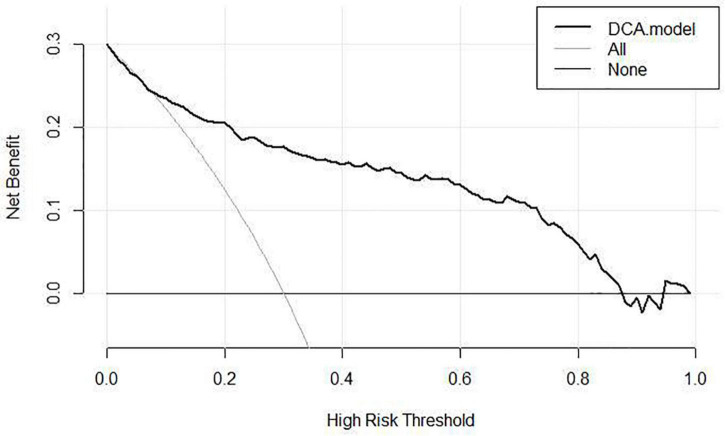
DCA of the nomogram.

As Figure 3 illustrates, the nomogram was validated using tenfold cross-validation, and the average area under the curve (AUC) was 0.862 (95% CI 0.816–0.909). This indicated that the nomogram was efficient in distinguishing between success and failure. At the same time, we obtained the calibration curve of the model with the mean absolute error of 0.021 (Figure 4), indicating that the actual prediction accuracy was close to the ideal prediction accuracy. The cutoff value for the model of 0.361 had a sensitivity of 70.6% and a specificity of 88.9%. Finally, the decision curve analysis (DCA) showed that clinical decisions could benefit by applying this online nomogram with the extent of the threshold (Figure 5).

## Discussion

Pavlik harness is the first choice for DDH in 0- to 6-month-old infants, and its overall therapeutic efficacy is satisfactory (18, 19). However, with the increased use of the Pavlik harness, there were more and more harness-associated drawbacks reported. On the one hand, complications of Pavlik harness include femoral nerve palsy, residual acetabular dysplasia, and avascular necrosis of the femoral head (AVN). A multicenter study in Japan included 4000 cases of total dislocation of the hip treated with Pavlik harness and found that the incidence of AVN was 11% (12). Kitoh found that the incidence of AVN was 8.8% (16/182) by following up 182 hips with successful Pavlik harness reduction for 1 year (3). Tiruveedhula found that the rate of AVN in closed reduction/spica casting treatment after failure of Pavlik harness was as high as 28%, which was significantly higher than the 8% in the direct closed reduction/spica casting treatment group (20). On the other hand, in the past years, clinicians have focused on risk factors of Pavlik harness failure, such as older age at the time of initiation (21–23), bilateral dislocation (3, 24), male (16, 21, 25), and Graf IV (6, 18). However, the above factors were mainly reported as single factor. Combining these predictors into a predictive model would allow for a better risk assessment and patient selection than single predictor or test. It is worth noting that since concomitant deformity is also a risk factor for treatment failure (3), children with concomitant deformities were also included in the study. At the same time, type IIa DDH was excluded which tends to have the potential to heal itself (17). The above factors may lead to a higher failure rate (27.4%, 102/372) of Pavlik harness compared to some previous studies.

After analyzing the remaining factors by univariate and multivariable logistic analyses, we developed the first online failure rate prediction nomogram for Pavlik harness based on age at initial treatment, concomitant deformity, and angle α. At the same time, our nomogram was evaluated by tenfold cross-validation, ROC analysis, calibration curves, and DCA, making the model more reliable. The model presented a predictive accuracy of 0.862 on ROC analysis, a good sensitivity of 70.6%, a good specificity of 88.9%, and a clinical net benefit in the range of probabilities between 5 and 90%, which has a certain clinical application value. Because the data on the above three aspects of the patient were conveniently acquired on an outpatient basis, the model could aid pediatric orthopedic surgeons to estimate the failure rate of Pavlik harness and make more effective clinical decision.

According to our previous study (26) and related literature (5, 15–17), there were increased success rates and better hip development for DDH treated by the human position brace or closed reduction/spica casting. However, for children with risk factors of treatment failure, Pediatric orthopedic surgeons still needed to try the Pavlik harness initially (17). If the Pavlik harness treatment failed, the treatment method would be changed to other strategies. But the treatment duration may be prolonged and the incidence of complications may increase (20). Now our model has important clinical implications in predicting the treatment outcomes of Pavlik harness in advance. For infants with a high probability of Pavlik harness failure, the clinicians enable to consider the human position brace and closed reduction/spica casting as an alternative, or even as the first choice to address the specific deficits of the Pavlik harness.

There were several limitations of our study. First, the accuracy of this model does not reach 100%. For children with a high failure rate, the harness could still be used after fully informing their parents of the possible outcomes, but these patients should be followed up closely. Second, possible risk factors of birth status (breech birth, oligohydramnios, etc.) and family history were not included in this study. However, these factors were not routinely required in clinical work and were rarely reported as risk factors for DDH treatment failure. Thus, the establishment of the model may be not strictly dependent on birth status and family history. Third, external validation of this new predictive model is required before its implementation in clinical practice and possibly using an app model presentation. A late-stage multicenter study with longer follow-up, applying to more races, will be conducted.

In our study, we developed the first prediction model based on simple and common clinical indicators, to predict the early failure rate of Pavlik harness in 0- to 6-month-old patients with developmental dysplasia of the hip. Because of its reasonable accuracy, this nomogram could be a useful resource for the orthopedic surgeon to identify and follow up patients at major risk of Pavlik harness failure who might need more reliable treatments.

## Data availability statement

The original contributions presented in this study are included in the article/supplementary material, further inquiries can be directed to the corresponding author.

## Author contributions

PG designed the study, drafted the original manuscript, and revised it. KG designed the data collection tool and gathered the data. XW conducted a preliminary analysis. XL was accountable for all aspects of the work in ensuring that questions related to the accuracy or integrity of any part of the work were appropriately investigated and resolved. All authors fulfill related requirements and accept the final submission.
